# Lectin histochemistry of posterior lingual glands of developing rats

**DOI:** 10.1038/s41598-023-36154-9

**Published:** 2023-06-26

**Authors:** Kazuma Harada, Koji Miki, Susumu Tanaka, Mikihiko Kogo, Satoshi Wakisaka

**Affiliations:** 1 The First Department of Oral and Maxillofacial Surgery, Graduate School of Dentistry, Osaka University, Suita, Japan; 2 Department of Periodonology, Graduate School of Dentistry, Osaka University, Suita, Japan; 3 Department of Anatomy and Cell Biology, Graduate School of Dentistry, Osaka University, Suita, Japan

**Keywords:** Structural biology, Anatomy

## Abstract

The posterior lingual glands are classified as Weber and von Ebner glands. Glycans play an important role in salivary glands. Although the distribution of glycans can explain functional diversity and variation, there are many unknowns in the developing rat posterior lingual glands. The purpose of this study was to elucidate the relationship between the development and function of the posterior lingual gland in rats by histochemical analysis using lectins that bind to sugar residues. In adult rats, *Arachis hypogaea* (PNA), *Glycine maximus* (SBA), and *Triticum vulgaris* (WGA) were associated with serous cells and *Dolichos biflorus* (DBA) with mucous cells. In both Weber's and von Ebner's glands, all 4 lectins were bound to serous cells in early development, but as development progressed, DBA disappeared in serous cells and only the DBA remained in mucous cells. These results suggest that Galβ (1,3) > Galβ(1,4) > Gal, αGalNAc > αGal > βGalNAc, NeuAc > (GalNAc)_2–3_>>>GlcNAc, and GalNAcα(1,3) are present in the early stage of development, but that GalNAcα(1,3) disappear in serous cells and only GalNAcα(1,3) are localized in mucous cells after maturation. These results indicate that Weber glands function as serous glands in the early postnatal stage when von Ebner glands have not matured.

## Introduction

The oral cavity is part of the digestive system whose main function is to aid food intake. The salivary glands produce and secrete saliva, which keeps the oral cavity moist. Lingual gland’s saliva is an important growth factor for taste receptor cells^1^. The tongue has two minor salivary glands, the anterior lingual and post lingual^2^. The anterior lingual gland, Blandin-Nühn's gland, is a mixed gland located on the inferior surface of the tongue apex^2,3^. In humans, Weber's glands open onto the tonsils of the tongue^2,4,5^, whereas in rats, Weber’s glands open onto the dorsal epithelium at the back of the tongue, and are involved in food mass formation and swallowing^2,5^. Weber's glands are muciparous; however, the presence of serous cells has been suggested in humans and rats^2,6^. The von Ebner’s glands, which open at the base of the sulcus of the circumvallate papilla and papillae foliate, are serous^2,7,8^ and secrete saliva and wash out the taste pits of taste buds in the papillary sulcus epithelium, thereby renewing and maintaining taste receptor function^6,9–12^. They also produce tongue lipase, which hydrolyzes triacylglycerols in the acidic gastric lumen and aids the first step in dietary fat digestion^2,13^. In rats, the development of the minor salivary glands occurs in the late embryonic period^5^, whereas the development of major salivary glands begins in the parotid gland on embryonic day 14^5,14,15^, in the submandibular gland on embryonic day 13^16^, and in the sublingual gland approximately on embryonic day 18^17^.

The posterior lingual glands are believed to be involved in the renewal of taste receptors through salivation and washing of the taste pits of the taste buds with saliva. Lectins are often used as markers for epithelial and mesenchymal cells to identify specific cell populations because of their ability to bind specifically to glycohydrate epitopes on the cell membrane. By searching for the binding mode of lectins, the localization of glycoconjugates in different tissues and the characteristics of cellular glycans in each tissue can be elucidated. Lectin histochemical studies have been reported on the distribution of lectins in various species. In summary, the previous reports indicate that serous cells of salivary glands show binding to *Ulex europeus* agglutinin-I (UEAI), Soybean *Glycine maximus* agglutinin (SBA), Peanut (*Arachis hypogaea*) agglutinin (PNA) and Wheat germ (*Triticum vulgaris*) agglutinin (WGA), while mucous cells tend to show binding to *Ricinus communis* agglutinin (RCA) and Horse gram (*Dolichos biflorus*) agglutinin (DBA)^18–31^. From the 21 lectins used in our laboratory and potentially binding to salivary glands, we found that SBA, PNA, and WGA bind to serous cells and DBA binds to mucous cells in adult rats.

However, although the localization and site differences of these lectin-binding patterns have been clarified, the development of the rat posterior lingual glands and changes in lectin-binding patterns have not been clarified. In this study, we examined the development of the rat posterior lingual glands and lectin-binding patterns using SBA, PNA, WGA, and DBA as indices, with the aim of clarifying the relationship between the development of the posterior lingual gland and the renewal of taste reception among its functions. The following results were obtained. Weber’s glands and von Ebner glands were fully developed by postnatal day 21. Mucous cells of the Weber’s glands were shown to bind DBA in adult rats, and SBA, WGA, PNA and DBA in the early developmental stage. Serous cells of von Ebner’s glands were shown to bind SBA, WGA, and PNA in adult rats, and DBA bound to the cellular membrane during early development but disappeared during development. Matured taste buds were found on the circumvallate papilla on postnatal day 1. The present findings indicate that the maturation of posterior lingual glands is closely associated with changes in food habit, and that the Weber’s gland functions as a serous gland in the early postnatal stage, while the von Ebner’s gland is not yet mature. The lectin-binding properties suggest the presence of Galβ (1,3) > Galβ(1,4) > Gal, αGalNAc > αGal > βGalNAc, and NeuAc > (GalNAc)_2–3_>>>GlcNAc sugar residues in serous cells and GalNAcα(1,3) sugar residues in mucous cells (Table 1). The results obtained provide data for the functional characterization of the rat posterior lingual gland.

**Table 1:** Four lectins used in this study and their binding Specificity. The four lectins used in this study for serous and mucus cells, their origins, abbreviations, and binding specificities are listed in table. Decreased specificity is represented by one or more greater symbols. Abbreviations: Gal, galactose; GalNAc, N-acetylgalactosamine; NeuAc, sialic acid; GlcNAc, N-acetylglucosamine.

Abbreviation	Lectin	Oligosaccharide specificity
PNA	Peanut (*Arachis hypogaea*) agglutinin	Galβ (1,3) > Galβ(1,4) > Gal
SBA	Soybean (*Glycine maximus*) agglutinin	αGalNAc > αGal > βGalNAc
WGA	Wheat germ (*Triticum vulgaris*) agglutinin	NeuAc > (GalNAc)_2–3_>>>GlcNAc
DBA	Horse gram (*Dolichos biflorus*) agglutinin	GalNAcα(1,3)

## Results

### Hematoxylin and Eosin (H&E) staining

**Figure 1:**
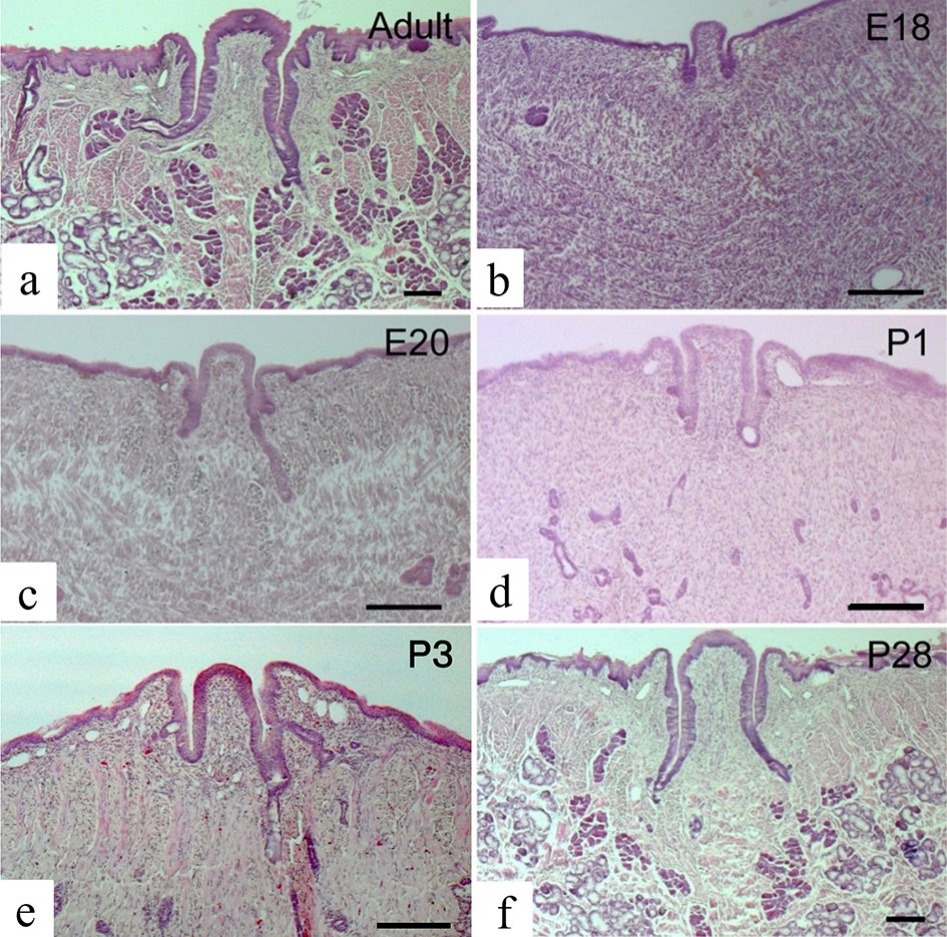
Hematoxylin and Eosin staining of a rat lingual frontal section containing a circumvallate papilla. Cross-sections of the rat tongue including the circumvallate papilla at postnatal day 56 (**a**), embryonic day 18 (**b**), embryonic day 20 (**c**), postnatal day 1 (**d**), postnatal day 3 (**e**), and postnatal day 28 (**f**). Scale is 200 µm. E: embryonic, P: postnatal.

In adult rats, von Ebner's glands were found to be located just below the circumvallate papilla, separated on both sides by the lingual septum, clumped within the muscularis, and with the opening at the base of the circumvallate papilla sulcus (Fig. 1a). The Weber's glands were located laterally and posteriorly to the von Ebner’s glands, opening directly onto the dorsal surface of the tongue (Fig. 1a). On embryonic day 18, the sulcus epithelium of the circumvallate papilla had not yet been inserted into the mesenchyme. Serial sections showed clusters of epithelial cells in the lateral portion of the posterior tongue (Fig. 1b) which were continuous with the surface layer of the tongue epithelium. On embryonic day 20, we observed an infiltration of the sulcus epithelium of the circumvallate papilla into the mesenchyme, with no evident formation of the von Ebner’s glands. The Weber's glands were also found in the mesenchyme in high number (Fig. 1c) with numerous clusters of epithelial cells present in the mesenchyme at 1 day of age (Fig. 1d), and their number and size increased further at 3 days of age (Fig. 1e). Thereafter, the cell population of epithelial cells in the mesenchyme increased over time, and both the Weber’s and von Ebner’s glands were fully developed by 28 days of age (Fig. 1f).

**Figure 2:**
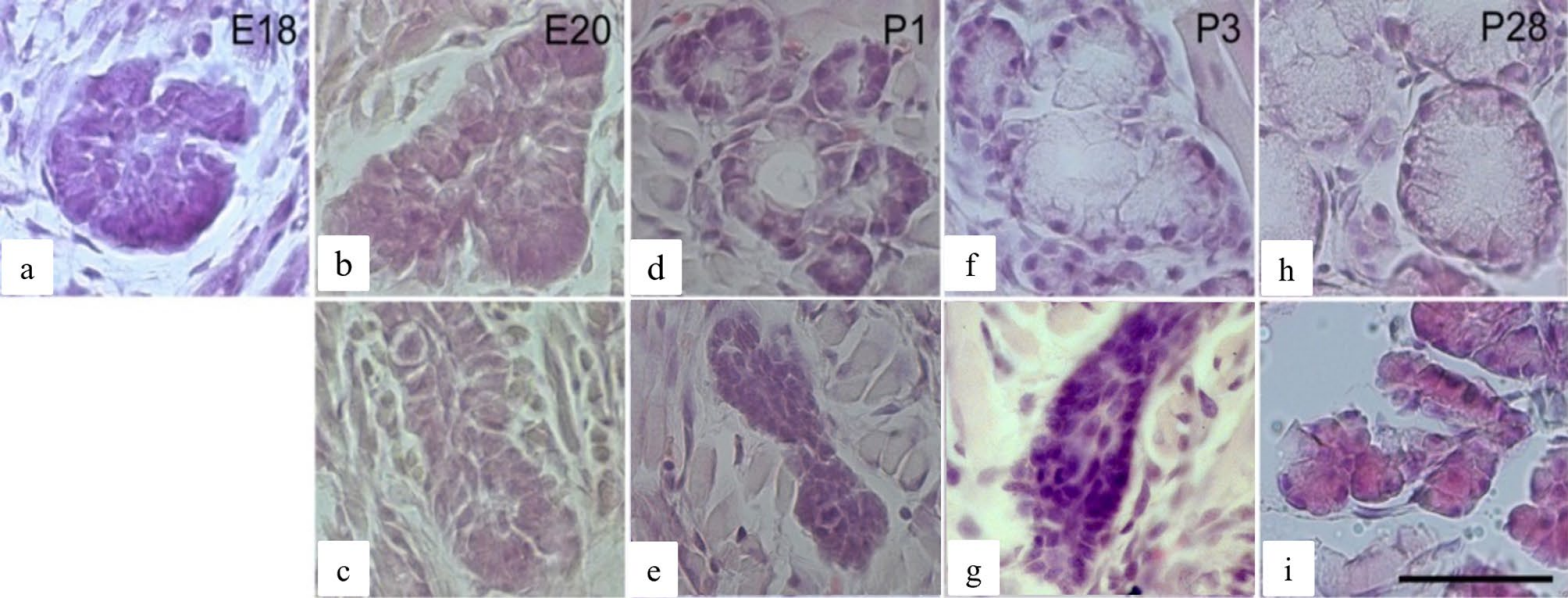
Hematoxylin and Eosin staining during development of terminal portion in the Weber's and von Ebner’s glands. Images of the Weber’s gland (**a**, **b**, **d**, **f**, **h**) and von Ebner’s gland (**c**, **e**, **g**, **i**) endings at embryonic day 18 (**a**), embryonic day 20 (**b**, **c**), postnatal day 1 (**d**, **e**), postnatal day 3 (**f**, **g**), and postnatal day 28 (**h**, **i**). Scale is 50 µm.

The clusters of epithelial cells observed at 18 days of age were formed by cells with darker cytoplasm (Fig. 2a). On embryonic day 20, the number of cells forming these cell masses increased, and the cytoplasm of these cells were similarly dark (Fig. 2b). No apparent formation of terminal cells of the serous glands at the tip of the sulcus epithelium of the circumvallate papilla, which were plunging into the mesenchyme on embryonic day 18 was observed (Fig. 2c). At 1 day of age, the terminal part of Weber's glands showed characteristics of mucous cells, with some cells having a light-toned cytoplasm as observed in adult rats, and others having dark-toned cytoplasm (Fig. 2d). Duct and epithelial mass were observed from the base of the papillae sulcus, some of which showed serous cell morphology and the formation of von Ebner’s glands (Fig. 2e).

Some of the Weber’s glands in rats aged 1 day showed flat nuclei in the clear-toned cytoplasm, and the number of these cells increased at 3 days of age. Serous demilunes were also observed (Fig. 2f). von Ebner’s glands showed tubular arrangements of terminal cells with round nuclei on the basal side of the dark-toned cytoplasm (Fig. 2g).

At 7 days of age, Weber's glands showed mucous cells, numerous serous cells, and a serous demilunes, whereas von Ebner’s glands demonstrated advanced branching of the terminal part of the gland**.**

Furthermore, Weber's glands showed a marked increase in mucous cells with age. At 21–28 days of age, the observations were similar to those noted in adult rats, and the cytoplasm of the terminal cells were transparent and filled with mucus secretions. The nuclei were oval and located basolaterally. Weber's glands contained mucous cells and serous cells with dark-toned cytoplasm (Fig. 2h).

The von Ebner’s glands showed increased terminal areas and branching, with triangular terminal cells surrounding the gland lumen, as seen in typical exocrine glands. In addition, round nuclei were located slightly basolateral to the basophilic cytoplasm (Fig. 2i).

**Figure 3:**
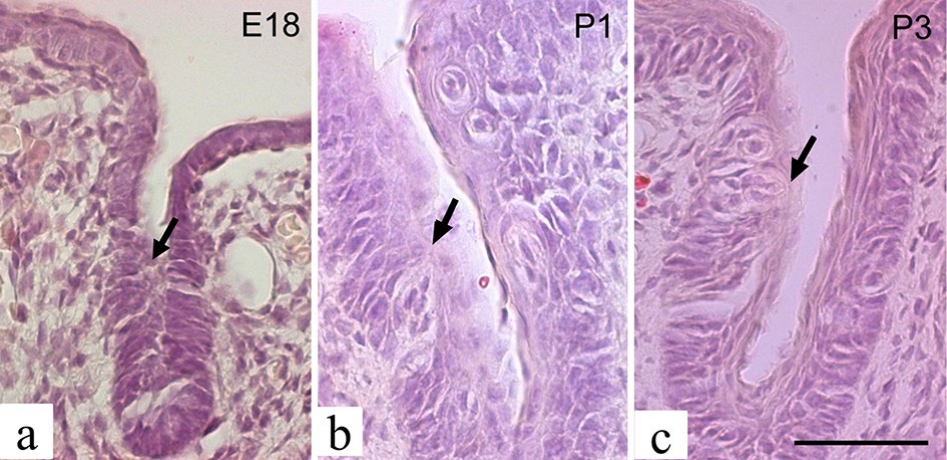
Hematoxylin and Eosin staining of the epithelium of the circumvallate papilla sulcus. Epithelium of the circumvallate papilla sulcus at embryonic day 18 (**a**), postnatal day 1 (**b**), and postnatal day 3 (**c**); arrows in (**a**, **b**, **c**) indicate the taste hole. Scale is 50 µm. E: embryonic, P: postnatal.

In the epithelium of the circumvallate papilla sulcus, taste bud primordia were present on embryonic day 18; however, no taste pits were observed (Fig. 3a). On postnatal day 1, taste buds with taste pits were observed (Fig. 3b), and thereafter the number of taste buds with taste pits increased (Fig. 3c).

### Lectin histochemistry

Among the lectins that have been reported to be used in salivary glands, in this study, we tested their binding in serous cells and mucous cells in the posterior lingual gland of adult rats. Three or more tissue fragments from each sample were validated with 21 different lectins. The binding of the lectins was classified into four levels: strong (3), moderate intensity (2), weak intensity (1), and unreactive (0). Different binding intensities were denoted by minimum and maximum values. The lectin that bind stongly to serous cells and unreactively to mucous cells are PNA, SBA, and WGA, which were used as markers for serous cells. The lectin that binds stongly to mucus cells and binds unreactively to mucus cells was DBA, which was designated as a marker for mucus cells. These 4 lectins were excellent markers for serous and mucous cells in adult animals, and were used as indicators of mature glandular cells and for serous and mucous cells during rat development (Table 2). Abbreviations: Con A, (*Canavalia ensiformis*) agglutinin; SBA, Soybean (*Glycine maximus*) agglutinin; WGA, Wheat germ (*Triticum vulgaris*) agglutinin; DBA, Horse gram (*Dolichos biflorus*) agglutinin; UEA-I, (*Ulex europaeus*) agglutinin-I; RCA-I, (*Ricinus communis*) agglutinin-I; PNA, peanut (*Arachis hypogaea*) agglutinin; GS-I, (*Griffonia simplicifolia*) Lectin I; pea, (*Pisum sativum*); LCA, (*Lens culinaris*) agglutinin; PHA-E, (*Phaseolus vulgaris*) erythroagglutinin; PHA-L, (*Phaseolus vulgaris*) leucoagglutinin; SJA, (*Sophora japonicum*) agglutinin, sWGA, Succinyl (*Triticum vulgaris*) agglutinin; GS-II , (*Griffonia simplicifolia*) Lectin II; DSA, Thorn apple (*Datura stramonium*) agglutinin; ECL (*Erythrina cristagalli*) Lectin; Jacalin, (*Artocarpus integrifolia*); LEL, (*Lycopersicon esculentum*) Lectin; STL, (*Solanum tuberosum*) Lectin; VVA, (*Vicia villosa*) agglutinin.

**Table 2:** Evaluation of lectin-binding properties of serous and mucous cells in adult rats. Staining intensity was evaluated and classified as 3: strong, 2: moderate intensity, 1: weak intensity, 0: unreactive. Variations in staining of the same structure were indicated by the minimum and maximum values of the staining range. The following 21 lectins were used. The following 21 lectins were validated in this study. Con A (*Canavalia ensiformis*), SBA (Soybean (*Glycine maximus*) agglutinin), WGA (Wheat germ (*Triticum vulgaris*) agglutinin), DBA (Horse gram (*Dolichos biflorus*) agglutinin), UEA-I ((*Ulex europaeus*) agglutinin-I), RCA ((*Ricinus communis*) agglutinin-I), PNA (peanut (*Arachis hypogaea*) agglutinin), GS-I ((*Griffonia simplicifolia*) Lectin I), pea ((*Arachis hypogaea*) agglutinin), LCA ((*Lens culinaris*) agglutinin), PHA-E ((*Phaseolus vulgaris*) erythroagglutinin), PHA-L ((*Phaseolus vulgaris*) leucoagglutinin), SJA ((*Sophora japonicum*) agglutinin), sWGA (Succinyl (*Triticum vulgaris*) agglutinin), GS-II ((*Griffonia simplicifolia*) Lectin II), DSA (Thorn apple (*Datura stramonium*) agglutinin), ECL ((*Erythrina cristagalli*) Lectin), Jacalin (*Artocarpus integrifolia*), LEL ((*Lycopersicon esculentum*) Lectin), STL (*Solanum tuberosum*) Lectin) and VVA ((*Vicia villosa*) agglutinin).

Lectin	Serous cell	Mucous cell
Con A	0–1	0
SBA	3	0
WGA	3	0
DBA	0	3
UEA-I	0	0
RCA	0	0
PNA	3	0
GS-I	0–1	0
pea	0	0
LCA	0	0
PHA-E	0–1	0
PHA-L	0	0
SJA	0	0
sWGA	0–2	0
GS-II	0–1	0
DSA	0	0
ECL	0–1	0
Jacalin	3	2–3
LEL	0	0
STL	0	0
VVA	2–3	3

### Lectin binding in the Weber’s glands

**Figure 4:**
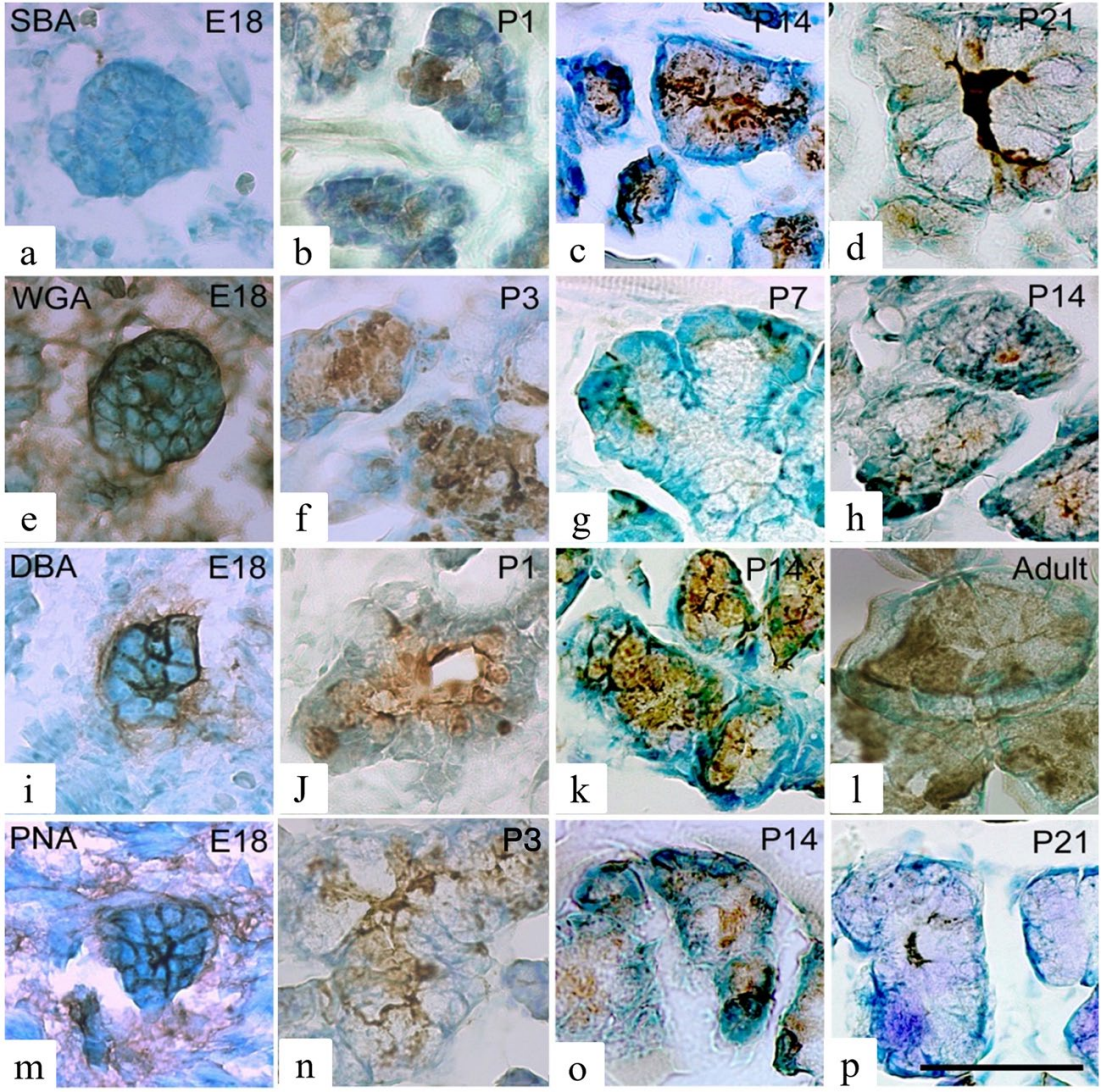
Lectin-binding patterns in the Weber’s glands at different ages. Binding patterns of SBA (**a**–**d**), WGA (**e**–**h**), DBA (**i**–**l**), and PNA (**m**–**p**) in the Weber’s glands at each age. Age is indicated in the upper right corner of each figure. Arrows indicate serous demilunes. Scale is 50 µm. E: embryonic, P: postnatal.

SBA binding to the cellular membrane was observed, albeit weakly, in cell masses on embryonic day 18 (Fig. 4a), and some SBA binding was noted in the cytoplasm on postnatal day 1 (Fig. 4b). At postnatal day7, SBA bound to the cytoplasm and cellular membrane, and at postnatal day14, SBA showed a similar binding pattern and bound to the serous demilunes (Fig. 4c). However, by postnatal day 21, the binding observed in the cytoplasm and cellular membrane was minimal (Fig. 4d). In the postnatal period, SBA binding was also strong in saliva. WGA bound to the cellular membrane of cells forming clusters on postnatal day 18, but not to the cytoplasm (Fig. 4e). WGA binding was observed in the cytoplasm, and from postnatal day 3, it was seen in the cell membrane (Fig. 4f); however, by postnatal day 7, both cytoplasmic and cell membrane WGA binding had disappeared. It was observed in serous cells (Fig. 4g). On postnatal day 14, WGA binding was observed in the serous demilunes, but not on the cellular membrane (Fig. 4h).

DBA exhibited strong binding to the cellular membrane on embryonic day 18 (Fig. 4i) and to the cytoplasm on postnatal day 1 (Fig. 4j). Thereafter, DBA bound to both the cellular membrane and cytoplasm; however, in adult rats, DBA binding to the cellular membrane decreased (Fig. 4k, l).

PNA bound to the cellular membrane from embryonic day 18 (Fig. 4m) and to the cytoplasm from postnatal day 3, and binding was observed until postnatal day 14 (Fig. 4n, o); however, by postnatal day 21, PNA binding to the cellular membrane was absent (Fig. 4p).

### Lectin binding in the von Ebner’s glands

**Figure 5:**
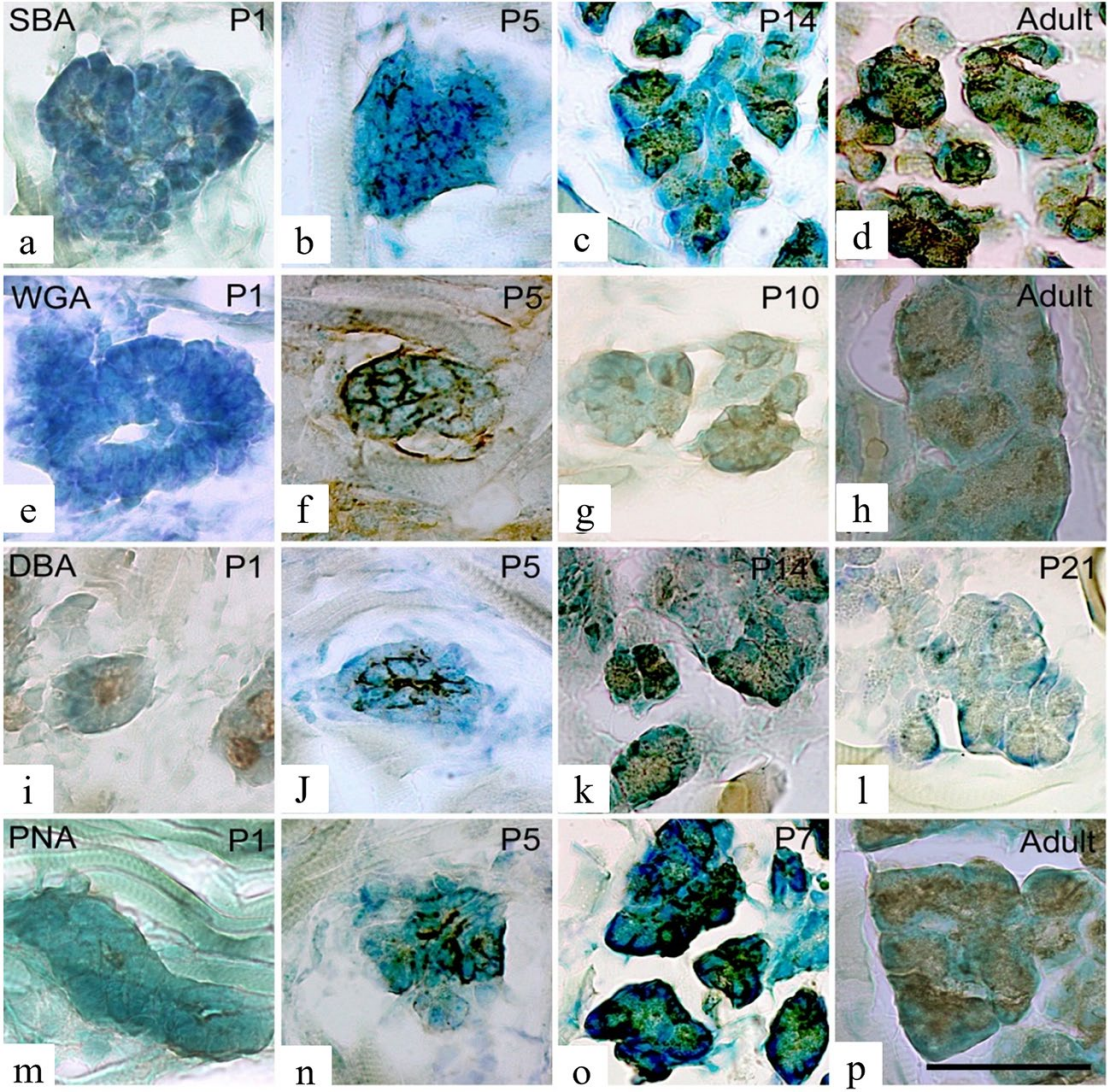
Lectin-binding patterns in the von Ebner’s glands at different ages. Binding patterns of SBA (**a**–**d**), WGA (**e**–**h**), DBA (**i**–**l**), and PNA (**m**–**p**) in the von Ebner’s glands at each age. Age is indicated in the upper right corner of each figure. Scale is 50 µm. E: embryonic, P: postnatal.

From postnatal day 1, when the terminal cell primordium of von Ebner’s glands was recognized, SBA binding was observed at the cellular membrane (Fig. 5a), and the binding remained constant with age (Fig. 5b–d). Binding was also observed in the cytoplasm from postnatal day14 (Fig. 5c). Adult rats showed SBA binding in the cytoplasm and on the cellular membrane (Fig. 5d).

WGA exhibited almost no binding at postnatal day 1 (Fig. 5e); however, at postnatal day 5, 10, WGA binding to the cellular membrane was strong (Fig. 5f, g). In adult rats, WGA binding was also observed in the cytoplasm (Fig. 5h). DBA binding was observed on the cellular membrane at postnatal day 1 (Fig. 5i), and the same binding pattern was observed until postnatal day14 (Fig. 5j, k). However, after postnatal day 21, no DBA binding was observed in either the cytoplasm or cellular membrane (Fig. 5l). PNA binding was identified on the cellular membrane at postnatal day 1 (Fig. 5m), which grew stronger with age (Fig. 5n, o). PNA binding was also observed in the cytoplasm of adult rats (Fig. 5p).

## Discussion

Here, the development of Weber's and von Ebner's glands, which are posterior lingual glands around the circumvallate papilla in rats, was searched histochemically and by lectin histochemistry.

In rodents, 4 types of papillae exist on the dorsal surface of the tongue: fungiform, circumvallate, foliate, and filiform papillae^32^. In humans, the papillae vallate are circular bulges with 8–9 papillae^4^. However, in mice and rats, a single circumvallate papilla exists on the posterior midline of the dorsal surface of the tongue. This circumvallate papilla contains taste buds^12,33^. In addition, the von Ebner’s glands open onto the base of the fornix and lobulated papillary sulcus, and the Weber's glands are present laterally and posteriorly to the von Ebner's glands. The Weber's glands are mucous glands^34,35^; however, the presence of serous cells has been suggested in humans, rats, and mice^31,34^. Here, the Weber's glands contained mucous cells and fewer serous and serous semilunar cells. These findings are consistent with previous findings in rats^8,34^, humans^2,36^, and other animals^2,8^.

In the present study, on embryonic day 18, the epithelial cells of the mesenchyme in the posterior part of the tongue were continuous with the tongue epithelium and could be considered the Weber's gland primordium. These cells showed serous cell morphology. Hamosh et al*.*^5^ reported that the development of the Weber's glands preceded that of the von Ebner’s glands, and secretory granules were present by embryonic day 20. These findings are in close agreement with those of the present study. Regarding the development of the palatine glands, which are considered to be muciparous like Weber's glands, Shinzato et al. ^37^*.* reported that in rats, epithelial incision began on embryonic day 17, terminal cells were formed on embryonic day 18, and mucous cells appeared on embryonic day 21. This change is almost identical to the development of the Weber’s glands demonstrated in the present study.

Here, von Ebner’s glands were not formed on embryonic day 18; however, from embryonic day 20, the epithelium of the circumvallate papilla sulcus started infiltrating into the mesenchyme. Hamosh et al*.*^5^ showed that in Sprague Dawley rats, the epithelial growth from the foliate and circumvallate papillae on embryonic day 19–20 was noted as the von Ebner’s glands began to develop, and no terminal formation or production of secretory granules were observed until 3–4 days after birth. The results of the present study are in close agreement with that of these findings.

Analysis of sugar residues related to lectin histochemistry is underway in various fields^30,38–44^. The lectins are often used as markers identify specific cell populations because they bind specifically to glycohydrate epitopes on the cell membrane. By searching for the binding mode of lectins, the localization of glycoconjugates in different tissues and the characteristics of cellular glycans in each tissue can be elucidated. We found that SBA, PNA, and WGA bind to serous cells and DBA binds to mucous cells in adult rats. The results showed that only DBA was found to bind to Weber's gland mucosa cells in adult rats, while all four lectins, SBA, PNA, WGA and DBA, bind to the cells early in development, and all but DBA disappear during development. On the other hand, serous cells of von Ebner's glands bound SBA, WGA and PNA in adult rats, but all four lectins, SBA, PNA, WGA and DBA, bound to the cytoplasm during the developmental stage, and DBA was lost during development. The lectin-binding mode of the developing rats was similar to that of adult rats at postnatal day 21. In addition, mature taste buds were observed in the circumvallate papilla on postnatal day 1. These results indicate that the maturation of the posterior lingual glands is closely related to changes in eating habits, that they are already accompanied by taste receptors in the early postnatal period, but that the Weber's glands, which are mucous glands, function as serous glands and compensate for their function during the period when the von Ebner’s glands are immature. Lectin-binding properties indicate that serous cells. The lectin-binding properties suggested the presence of Galβ (1,3) > Galβ(1,4) > Gal, αGalNAc > αGal > βGalNAc, NeuAc > (GalNAc)_2–3_>>>GlcNAc sugar residues in the serous cells and GalNAcα(1,3) sugar residues in mucous cells.

Histochemically, Weber's glands, which are mucous glands, appeared as a cell mass and had the morphology of serous cells at embryonic day 18. At the same time, PNA, SBA, WGA, and DBA showed binding properties to the cellular membrane in lectin. This suggests that the sugar residues are Galβ (1,3) > Galβ(1,4) > Gal, α-D-GalNAc;β-D-GalNAc, NeuAc > (GalNAc)_2–3_>>>GlcNAc , and GalNAcα(1,3). As the Weber's gland matured, mucous cells appeared at postnatal day1, coinciding with this period of SBA and DBA binding. At this time, the cytoplasm of the mucus cells contained sugar residues of αGalNAc > αGal > βGalNAc and GalNAcα(1,3), and a little later, on postnatal day3, binding was observed to PNA and WGA, suggesting the presence of Galβ (1,3) > Galβ(1,4) > Gal and NeuAc > (GalNAc)_2–3_>>> GlcNAc in addition to these The presence of Galβ (1,3) > Galβ(1,4) > Gal and Β-D-GlcNAc;NeuNAc was suggested. Furthermore, WGA disappears on postnatal day 7 and PNA and SBA stop binding on postnatal day 14, suggesting that NeuAc > (GalNAc)_2–3_>>>GlcNAc disappears on postnatal day 7, Galβ (1,3) > Galβ(1,4) > Gal andαGalNAc > αGal > βGalNAc disappear on postnatal day 14, and only GalNAcα(1,3) residue may be present, respectively. Thus, it is suggested that the localization of sugar residues in the cell may change during development.

The loss of lectin binding coincided with the change in food intake from a liquid to a solid diet.

The salivary glands of rats aged 12–14 weeks and found that SBA, DBA, and PNA showed strong binding to serous cells of the posterior lingual gland, and SBA and DBA showed moderate binding to mucous cells. Further, in the serous semilunar cells found in mucous cells, the lectin-binding pattern was similar to that of serous cells. The authors also reported that SBA, DBA, and PNA showed moderate binding in serous cells, while the parotid gland, which is also serous in nature, showed weak binding in conduits^18^. These findings for DBA and PNA are consistent with those of the present study; however, the results regarding SBA are different. WGA also highlighted reactions in many of the serous terminal cells of the posterior lingual gland, as well as in mucous cells. Furthermore, PNA reacted with most of the serous terminal cells and only with serous cells in areas with a mixture of mucous and serous cells, but not in the conduit epithelium of the mucous glands.

These findings on lectin binding differ in part from the results of the present study. Lectins continue to bind to the same carbohydrates regardless of species, but the differences in binding properties may be due to differences in the carbohydrates present. The differences in binding patterns of lectin in methodology may also account for the discrepancy. The methodological difference is that some lectins are inactivated by phosphate complexed with Ca2 + , which causes excessive darkening of the tissue fragments and makes them extremely difficult to observe, so PBS was used for lectin staining in this study.

Here, terminal cells in the Weber's glands on embryonic day 18 and the von Ebner's glands on postnatal day 1 bound both serous cells and mucus cells before the lectin-binding pattern became identical to that of adult animals. This suggests that the Weber’s glands in late embryonic stages initially assume serous properties before forming mucous cells. It is possible that both the von Ebner’s and Weber’s glands function as mixed glands for some time after the onset of posterior lingual gland formation in the late embryonic period.

In the Weber's glands in rats, undifferentiated cells appear first and form serous cells that differentiate into intermediate-type cells, then into mucous cells. In the present study, we propose that the change in lectin-binding properties observed in the Weber's glands corresponds with this phenomenon.

In our study, we found that the posterior lingual gland in rats aged 21–28 days showed the same lectin-binding pattern as that of adult animals. In serous cells, αGalNAc > αGal > βGalNAc residues appeared at postnatal day14, suggesting that the salivary glands were also mature at this time, postnatal days 21–28, when the individuals matured, and Galβ (1,3) > Galβ(1,4) > Gal and NeuAc > (GalNAc)_2–3_>>>GlcNAc may be present. The timing of the appearance of these sugar residues may express the maturity of the developmental stages. Mucus cells may also be salivating and functioning on postnatal day1, when GalNAcα(1,3) residues are present. Furthermore, rats were weaned at postnatal day 21 and kept on solid feed thereafter. Thus, the change in diet may have caused a change in the properties of salivary gland cells. After postnatal day 10, the incisors erupt, and the rats can ingest solid food. During this period, the von Ebner’s glands secrete serous saliva and Weber's glands secrete mucous saliva. Mucous saliva is involved in food mass formation, and such changes in feeding behavior are thought to be closely related to changes in salivary components, i.e., the function of salivary secretory cells. Future studies should investigate these changes in weaning time and feed.

The von Ebner’s glands secrete serous saliva, which cleans the taste pits of the taste buds in the sulcus epithelium of the papillae foliate and circumvallate papilla thereby maintaining and renewing taste receptors^12,36^. Here, morphologically mature taste buds with taste pits were observed in the sulcus epithelium of the circumvallate papilla on postnatal day 1, and the number of these buds increased thereafter. The appearance of mature postnatal taste buds in the epithelium of the papillary sulcus is consistent with the findings of previous reports^45,46^. By postnatal day 3, The von Ebner's glands opening to the floor of the papillary sulcus was not mature; however, the Weber's glands opening to the mucosa behind the tongue showed morphological and histochemical characteristics of serous cells, suggesting that they secrete serous saliva. Therefore, until the von Ebner’s glands mature, Weber's glands are thought to renew taste receptors by cleaning the taste pits of the taste buds in the papillary sulcus epithelium, compensating for the function that the von Ebner’s glands perform. Similar changes may occur in humans, the same mammal species.

Not only do the secretory products of the von Ebner’s glands influence the taste response, but also the glyco-chemical properties of each taste bud^47,48^. We identified cases in which lectins bound to the cellular membrane and cytoplasm. The binding of lectins on the cellular membrane is thought to be due to the sugar chains that make up the cellular membrane. In contrast, lectin-binding in the cytoplasm is thought to be due to the binding of sugars to proteins formed intracellularly, which are modified by intracellular organelles. Here, we did not examine the intracellular aspects of lectins; however, future studies should examine this.

## Conclusion

**Figure 6:**
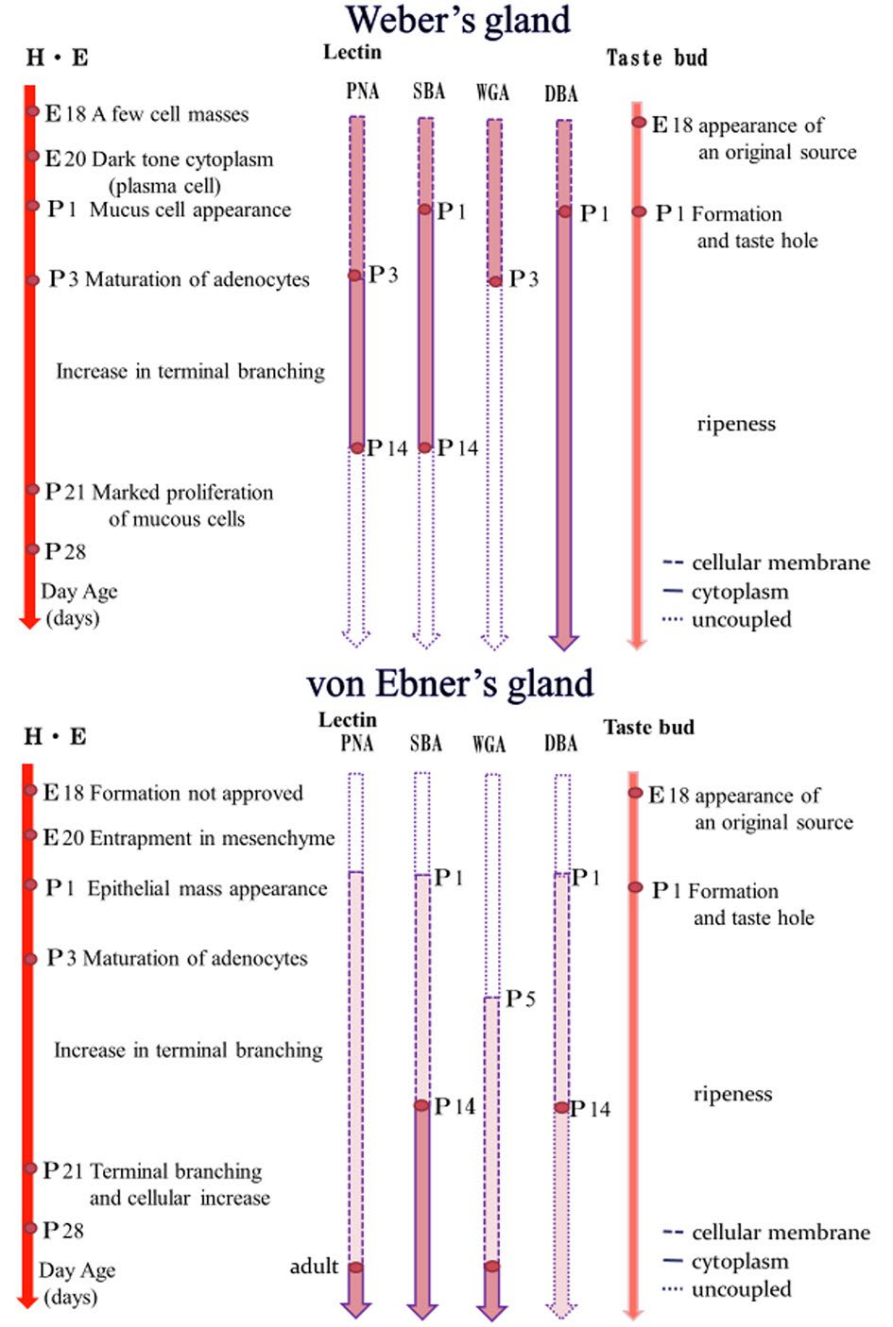
The development of Weber's and von Ebner’s glands was examined using lectin histochemistry using H&E staining, SBA, WGA, DBA, and PNA from the late embryonic stages of the rat. We identified that Weber's glands were a mass of serous cells on embryonic day18, followed by mucous cells postnatal day 3, and the number of mucous cells gradually increased with age, showing similar morphology to those of adult animals on postnatal day 21. Further, von Ebner glands were identified from about the 20th day of fetal life with the sulcus epithelium of the circumvallate papilla falling into the mesenchyme, but serous cells were not observed until postnatal day 1. Additionally, in the posterior lingual glands of adult rats, SBA, WGA, and PNA were found in serous cells, and DBA in mucous cells. E: embryonic, P: postnatal.

The development of the Weber's and von Ebner’s glands from the late embryonic stages of the rat were examined using lectin histochemistry using H&E staining, SBA, WGA, DBA, and PNA. We identified that the Weber's glands were a mass of serous cells on embryonic day 18, which formed mucous cells on postnatal day 3, and the number of mucous cells gradually increased with age, showing similar morphology to that of adult rats on postnatal day 21. Further, the von Ebner’s glands began to form around embryonic day 20; however, serous cells were not observed until postnatal day 3. Additionally, in the posterior lingual glands of adult rats, SBA, WGA, and PNA were found in serous cells, and DBA in mucous cells (Fig. 6). During early development, the Weber's and von Ebner’s glands showed both serous and mucous cell lectin-binding patterns, which were different from the lectin-binding patterns observed in adult rats. These findings suggest that the Weber's and von Ebner’s glands may function as mixed glands for a short period after the onset of posterior lingual gland formation during the postnatal period. Since developing rats showed the same lectin-binding pattern as adult rats from postnatal day 21–28, we suggest that their saliva secretion changed as their diet changed from liquid to solid.

## Materials and methods

### Experimental animals

At least three Sprague–Dawley rats of each age (embryonic day 18 and 20, and postnatal days 1, 3, 5, 7, 10, 14, 21, 28, 43, 56, and 63) were used. The day on which the vaginal plug was identified was defined as embryonic day 0, and the day of birth as postnatal day 0. Animals were weaned at postnatal day 21. For animals in the fetal period, pregnant rats were laparotomized under deep anesthesia administered intraperitoneally, and the fetuses were harvested and dissected. The specimens were fixed using immersion fixation. After birth, rats were placed under deep anesthesia administered intraperitoneally. The chest was opened, a catheter was inserted into the apex of the animal's heart and advanced to the base of the vena cava, an incision was made in the right ear, and the animal was immersed in 0.02 M phosphate-buffered saline (PBS; pH 7.4). The head was perfused and fixed in 0.1 M phosphate buffer (pH 7.4) containing 4% paraformaldehyde, and the tongue was removed and immersed in the same fixative solution. The rats were fed solid feed ad libitum. All animal experiments were approved by the Institutional Animal Care and Use Committee of Osaka University Graduate School of Dentistry and complied with the guidelines for the care and use of laboratory animals at Osaka University (Approval No. 19–024-0).

All animal experiments complied with the ARRIVE guidelines.

### Hematoxylin & Eosin staining

The posterior part of the tongue, including the circumvallate papilla, was paraffin-embedded and serial sections of 10 μm thickness were prepared. The paraffin sections were stained with hematoxylin and eosin (H&E) stain, and the morphological changes in the circumvallate papilla, Weber's gland, and von Ebner’s gland were observed under a light microscope.

### Lectin histochemistry

The posterior part of the tongue, including the circumvallate papilla, was embedded in optimal cutting temperature compound (Sakura Finetek Co., Tokyo, Japan), and 10 μm thick frozen sections were prepared and affixed to MAS–coated glass slides (Matsunami Glass Ind., Ltd., Osaka, Japan). The sections were washed with 0.02 M PBS, treated with 3% hydrogen peroxide-PBS for 30 min to inactivate endogenous peroxidase, washed with 0.02 M PBS, and incubated with biotin-labeled.

Con A, SBA, WGA, DBA, UEA-I, RCA-I, PNA, GS-I, pea , LCA, PHA-E, PHA-L, SJA, sWGA, GS-II, DSA, ECL, Jacalin, LEL, STL and VVA (0.5 μg/ml; Vector, Burlingame, CA, USA) in a wet box for 16 h at room temperature. The cells were then washed with 0.02 M PBS and treated with Avidin–Biotin Complex (Vector) for 90 min. We searched for the above 21 lectins in 3 sections per adult rat with N = 7 or greater. We performed lectin histochemical searches on the posterior lingual glands of rats. In the posterior lingual gland of rats, except for 4 lectins, some lectins showed binding to both serous and mucous cells, some did not show binding to both cells, and some rats showed unstable binding depending on the individual rats (Table 2). Their lectins bind strongly to serous cells and unreactively to mucus cells. PNA, SBA and WGA, were used as markers for serous cells. The lectin that binds strongly to mucus cells and binds unreactively to serous cells was DBA, which was used as a marker for mucus cells in this study. Sections were washed with 0.02 M PBS and further washed with 0.05 M Tris–HCl-buffered saline (TBS), pH 7.6, followed by 0.04% 3,3-diaminobenzidine (Sigma-Aldrich Co., Tokyo, Japan), and horseradish peroxidase activity was visualized using 0.05 M TBS containing 0.003% hydrogen peroxide water, and sensitized with 0.1% nickel ammonium sulfate. All reactions were performed at room temperature. After the reactions, the cells were contrast stained using methylene blue, dehydrated in ascending ethanol series, permeabilized with Lemosol, sealed in Permount ® (Fisher Scientific, NJ), and observed under an optical microscope^49–51^.

## Data Availability

All data generated or analysed during this study are presented in this paper. All the raw data files are available from the corresponding authors on reasonable request.
